# Multi-Channel Convolutional Neural Networks Architecture Feeding for Effective EEG Mental Tasks Classification

**DOI:** 10.3390/s18103451

**Published:** 2018-10-14

**Authors:** Sławomir Opałka, Bartłomiej Stasiak, Dominik Szajerman, Adam Wojciechowski

**Affiliations:** Institute of Information Technology, Łódź University of Technology, 90-924 Łódź, Poland; slawomir.opalka@edu.p.lodz.pl (S.O.); bartlomiej.stasiak@p.lodz.pl (B.S.); dominik.szajerman@p.lodz.pl (D.S.)

**Keywords:** mental task classification, EEG, CNN, BCI

## Abstract

Mental tasks classification is increasingly recognized as a major challenge in the field of EEG signal processing and analysis. State-of-the-art approaches face the issue of spatially unstable structure of highly noised EEG signals. To address this problem, this paper presents a multi-channel convolutional neural network architecture with adaptively optimized parameters. Our solution outperforms alternative methods in terms of classification accuracy of mental tasks (imagination of hand movements and speech sounds generation) while providing high generalization capability (∼5%). Classification efficiency was obtained by using a frequency-domain multi-channel neural network feeding scheme by EEG signal frequency sub-bands analysis and architecture supporting feature mapping with two subsequent convolutional layers terminated with a fully connected layer. For dataset V from BCI Competition III, the method achieved an average classification accuracy level of nearly 70%, outperforming alternative methods. The solution presented applies a frequency domain for input data processed by a multi-channel architecture that isolates frequency sub-bands in time windows, which enables multi-class signal classification that is highly generalizable and more accurate (∼1.2%) than the existing solutions. Such an approach, combined with an appropriate learning strategy and parameters optimization, adapted to signal characteristics, outperforms reference single- or multi-channel networks, such as AlexNet, VGG-16 and Cecotti’s multi-channel NN. With the classification accuracy improvement of 1.2%, our solution is a clear advance as compared to the top three state-of-the-art methods, which achieved the result of no more than 0.3%.

## 1. Introduction

A brain–computer interface (BCI) enables communication between the brain and external systems by means of messages and commands originating from users’ thoughts rather than from the physical activity of body parts or the operations of dedicated electronic controllers [1]. Various methods have been developed and introduced to record and interpret the electrical activity of the brain, e.g., magnetoencephalography (MEG), functional magnetic resonance imaging (fMRI), positron emission tomography (PET) and cortical evoked potentials monitored with electroencephalography (EEG). Since EEG is non-invasive, relatively inexpensive and convenient to acquire as compared to other signal acquisition methods, it has become especially popular in the development of affordable BCI systems, both stationary (for more accuracy-demanding tasks such as game control [1]) and mobile (for the flexibility of user motor activity in vehicle control [2,3,4]).

The major problems faced during semantic EEG signal analysis (mental tasks classification) are connected with the classification accuracy. EEG signal is recorded in the form of a multi-frequency band, usually affected by noise, due to the sensitivity of the recording equipment to external artifact sources, i.e., any devices generating electromagnetic fields, and susceptibility of the signal to distortion due to the rapidly changing dynamics of adjacent cortical areas activity. Recent solutions proposed in the literature rely on EEG signal factorization using time-domain data stream windowing and classification with convolutional neural networks [5,6,7,8,9]. Research has tended to focus on single-channel neural networks and their architecture, with experiments concerning mostly the number of layers, kernel characteristics affected by input data stream or the learning strategy [5,6,7,8,10,11,12]. Only Cecotti experimented with a multi-channel convolutional neural network architecture, dedicating the network channels to different time domain windows for signal analysis [9].

Surprisingly, none of the authors experimented with multi-channel neural network fed with frequency-domain signal sub-bands characteristics. The present study seeks to address this research gap. The solution presented, underpinned with an appropriate learning and optimization strategy, may enable the extraction of semantically important signal characteristics from the noised signal spectrum, offering a considerable potential for boosting classification accuracy.

The main contribution of this paper is a novel approach to multi-channel convolutional neural network architecture feeding for cortical evoked potentials-based mental tasks classification. Using a multi-channel CNN architecture fed with frequency-domain data divided into sub-bands rather than time-domain raw EEG signal, our solution provides the ability to use fewer EEG electrodes, thus offering a high degree of flexibility when it comes to the testing equipment set up, while also improving the classification results. At the same time, the approach presented enables multi-class classification, which also distinguishes it from other mental task-based solutions using CNNs.

Our previous work concentrated on developing a preliminary multi-channel architecture [13]. However, the previous solution suffered from a number of flaws. This study sought to eliminate these shortcomings by modifying the order and type of inner layers and providing additional comparisons with competitive convolutional architectures. Moreover, we experimented with kernel sizes, selected learning rate modification functions and learning strategies, which resulted in classification accuracy improvement. The accuracy tests were performed on the well-established and reliable experimental dataset V from BCI Competition III [14,15]. Our solution, fed with data according to the scheme presented, demonstrates a very high accuracy and noticeable generalization capability: the difference between classification accuracy during the learning process and dataset testing on average reached about 5%. Additionally, the final accuracy results obtained by the proposed method outmatched the results achieved by other neural network (NN)-based solutions, including the current BCI Competition III winner, well-known single-channel architectures (AlexNet and VGG-16 [12,16]) and the most relevant multi-channel network proposed by Cecotti [9].

## 2. State of the Art

The process of EEG signal interpretation in communication and control tasks typically consists of two stages: the extraction of signal features and the classification of resulting signal feature patterns.

The former may be approached using such methods as common spatial pattern (CSP), extreme energy ratio (ERR), orthogonal parametric transforms [17], autoregressive (AR) parameters, wavelet packet transform (WPT) [18], principal component analysis (PCA or KLT) [19,20] or hidden Markov model (HMM) to carry out dimensionality reduction [21]. All aforementioned methods are constantly modified to meet the growing usability and effectiveness requirements. However, nonlinear classifiers, such as convolutional neural networks (CNN), generally read in a raw, time-domain, windowed signal as input data [5,6,7,8,9]. Its aggregated, multi-frequency (from a few Hz to 200 Hz) form hinders the interpretation of a highly noised EEG signal. This paper endeavours to demonstrate that splitting frequency-domain data into sub-bands, combined with an appropriate network feeding scheme, provides the ability to retrieve semantically invaluable key signal characteristics from an aggregated EEG signal, while also increasing the network’s efficiency potential. More details on this are given below.

The latter stage of EEG signal processing involves the application of a classifier. A well-established technique in this field is linear discriminant analysis (LDA) [22], which separates data representing different classes by a hyperplane. Another group of methods for between-classes hyperplane construction are the support vector machines (SVM) [23,24,25]. Other approaches employ the Bayesian classifier [26], which assigns a feature vector to the class, or the Gaussian mixture model—a clustering method which rests on using the probability density function [27]. Another method of classification is provided by the steady state visual evoked potential (SSVEP), proposed as a trade-off solution among accuracy, responsiveness and complexity [28]. It is also possible to find a relation between some of the mental disorders and the brain neural network activity, which can be detected, for example, by the Phase Lag Index [29] or DWT, sample entropy and O_CCA [30].

One of the commonly applied classification approaches involves the use of the artificial neural networks (ANN)—a well-established tool for solving pattern-recognition problems. They are frequently used as classifiers of EEG signals in brain computer interfaces. A popular class of artificial neural network is the multi-layer perceptron (MLP). However, because they are universal approximators, MLP networks are vulnerable to overtraining, especially for non-stationary data such as EEG [22].

Recent studies have shown a growing interest in the Convolutional Neural Networks (CNN)—a concept inspired by the structure of the primate visual cortex [31]. The CNN architecture is based on a simple rule that only some of the following layer neuron inputs are connected with the output of the previous layer [16]. Apart from image classification [16,32], this type of neural network architecture proved to be successful in dealing with many other problems, such as sound signal analysis [33,34], as well as in medical applications [35].

The efficiency of CNNs inspired researchers to investigate their applicability to the classification of EEG signal recorded as the multi-dimensional cortical-evoked potential vector. Several functional problems have recently been approached using CNN-based EEG classification:

- imagined and/or executed movement [5,6,7,8,36,37,38];
- oddball response [7,9,10,39,40];
- epileptic seizure prediction/detection [41,42,43,44]; and
- other mental tasks, such as:
  – memory performance [45];
  – driver performance [46] and fatigue [47];
  – memorizing [11]; and
  – music-related tasks [48,49].

As opposed to some of the above-mentioned works, which investigated more than one functional problem, the present study concentrates on one, namely imagined limb movements. The paper investigates the use of CNNs in a new application area—imagined speech sound generation. This is discussed in more detail in the Section 3.

The performance of a CNN is closely related to its architecture design. Determining a proper architecture for a selected EEG functional classification problem involves such parameters as the number of layers, the number of channels and the filter size, as well as operational decisions concerning the structure of input features, the learning method and the optimization solvers.

A major challenge is to determine the appropriate depth of the network. While most researchers use 1, 2, or 3 convolution layers [6,7,8,9,36,37,38,39,40,41,42,43,44,45,46,48,49], some authors considered such architectures as “shallow” and proposed 4 [10], 5 [5], 7 [11] or even 19 layers [12]. However, the existing studies fail to provide a precise rule for the problem-related selection of the number of layers.

Previous works employed single-channel network architectures with numerous parameters and hundreds of thousands neurons in CNNs [16] and deep (up to 19 layers) CNN architectures [12]. The proposed method is distinct from the existing solutions in that it employs a multi-channel architecture with a novel organization of convolutional layers depending on the structure of input data.

As demonstrated in the relevant literature, network data feeding is a crucial factor for classification efficiency. Most of the researchers feed artificial networks with raw data in the time domain, especially when investigating the imagined movement classification problem [5,6,7,8,9]. The length of the time window varies across different studies—from several hundreds of milliseconds (for stimuli onset or offset detection) to time windows encompassing all stimuli.

In light of the above considerations, the only relevant example of a multi-channel network is the CNN proposed by Cecotti [9]. Dedicated to P300 detection, this solution applies time domain input data, with a single channel to the first convolutional layer containing a single time sample for the whole set of 64 EEG electrodes. At the same time, it serves to solve a single-class problem, which determines the architecture of its last layers. Cecotti’s method is not applicable to more demanding input data and does not work in cases where multi-class results are expected.

Likewise, Yang included sub-band frequency information, but the use of ACSP before processing in the neural network and the additional feature map selection algorithm increases the need for calculations [50]. This is a typical example of a CNN architectural solution with the convolutional layers interleaved with subsampling ones. Moreover, to make the classification task easier, the EEG data used for this experiment are additionally supported by electrooculographics data streams.

Although EEG signals can be analyzed in time and frequency domains, none of the aforementioned studies have favored any of these ways with respect to the decoding problem and none of the authors have experimented directly with convolutional neural network feeding with frequency-domain signal sub-bands. At the same time, as verified in this study, feeding the input in the frequency domain and signal factorization (frequency sub-bands splitting) may considerably affect the network’s classification efficacy. As typical frequencies consider a range from a few Hz to tens of Hz (sometimes up to 200 Hz), we divided the signal into twelve frequency sub-bands. This process is described in detail in the Section 3.

Each physiological signal acquired is vulnerable to environmental noise or distortions generated by the organism of the subject under examination. Thus, it is a common practice to remove artifacts from EEG and fMRI recordings. This can be performed using various methods, for example ICA and FastICA [51,52,53]. However, as this study considers raw EEG signal from the considered dataset, no preliminary signal processing procedure has been applied.

Upon setting the format of input data, the CNN designer does not have much freedom to manipulate the architecture. However, the networks usually differ in design parameter characteristics.

In the context of BCI systems, convolutional neural networks employ the learning process enhancement algorithms, e.g., back-propagation of errors (BPNN), genetic algorithms (GANN), particle swarm optimization (PSO) [54] or backtracking search optimization algorithm (BSANN) based on evolutionary algorithms [19]. Some of the enhancements are extended further, such as PSONN to Improved PSONN (IPSONN), which relies on the Modified (by migration) Evolutionary Direction Operator (MEDO) [19].

Other important aspects of CNN design that may exert a significant influence on the signal analysis accuracy include the type of activation functions (e.g., ELU and ReLU), the pooling mode (e.g., max and mean) and the splitting of convolution into spatial and temporal parts [5]. The design choices made in this study are explained in the Section 4.

To increase the learning speed and accuracy, it seems necessary to employ modern stochastic optimization solvers: AdaGrad [55], SGD [56], AdaDelta [57] and Adam [58]. Their significance for our research is discussed in Section 5.3.

## 3. Dataset

To compare the performance of various classification methods, it is necessary to rely on a standard reference task. The proposed solution was validated with tests performed on dataset V from BCI Competition III [14,15], which is recognized within the research community as a reliable procedure for an unbiased assessment of alternative methods. Initially, the best BCI competition solution was not based on a neural network, but on statistical discrimination with online discrimination improvement [59]. The algorithm achieved an average accuracy of 68.64%. More recent NN-based solutions show marginally better results under different assumptions. For example, Bhattacharya et al. used cross-validation instead of a test set for effectiveness calculation [60].

The dataset encompasses data from three EEG experiment subjects who were assigned three tasks during the data acquisition:

- the imagination of repetitive left hand movements (Class 2);
- the imagination of repetitive right hand movements (Class 3); and
- the generation of words beginning with the same random letter (Class 7).

**Figure 1:**
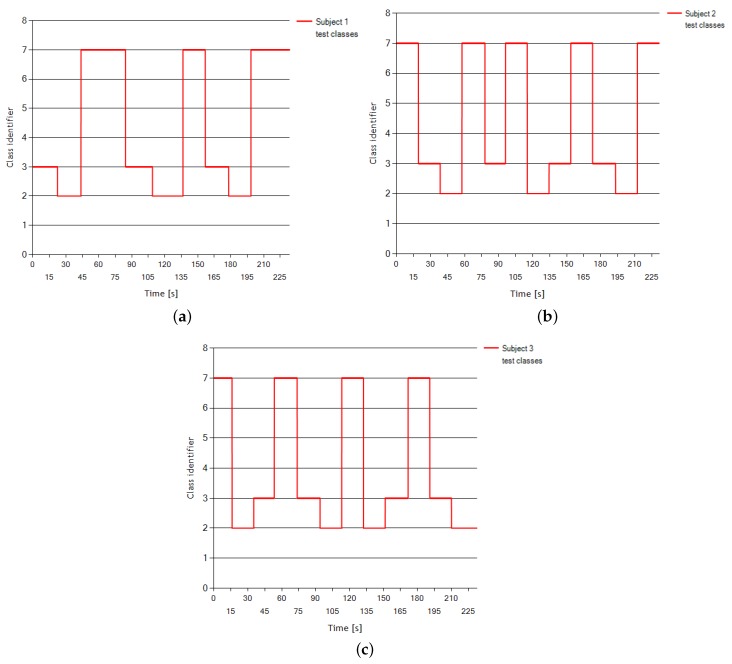
(**Top**) Mental tasks classes for Subject 1 (**a**) and Subject 2 (**b**) test sessions; and (**c**) mental tasks classes for Subject 3 test session.

The data were collected during four sessions for each subject. Each session lasted around 4 min during which the subject was asked to randomly switch between mental tasks every 15 s at the operator’s request. Between every session, there was a 5–10 min break. Figure 1 presents the change of classes over the test sessions—the last one of four—for every subject.

The dataset was collected using eight centro-parietal electrodes (C3, Cz, C4, CP1, CP2, P3, Pz, and P4 in the standard 10–20 placement system) with the sampling rate of 512 Hz. The raw EEG potentials were first spatially filtered using a surface Laplacian (http://www.bbci.de/competition/iii/desc_V.html). The single data record was saved every 62.5 ms (16 times per second) with the power spectral density estimated from raw EEG in the 8–30 Hz band (12 frequency sub-bands for each electrode with a frequency resolution of 2 Hz).

The works based on this dataset (e.g., [19,59]) typically do not apply any direct methods of data improvement and the dataset itself carries the information that it has not been subject to any artifact rejection or correction procedure.

It is worth mentioning that none of the previous solutions tested on this dataset, such as GANN, BPNN, PSONN, IPSONN and BSANN, was based on CNN.

## 4. Method

The method proposed in this manuscript differs significantly from similar solutions in the following aspects:

- It separates CNN processing into isolated channels, between which there is no data flow, until the fully connected layer.
- The two convolutionary layers in each channel are directly connected without isolating them with the subsampling layer; all typical CNN solutions interweave each CNN layer with a subsampling layer.
- Input domain for the data is frequency with its super sampling into 12 sub-bands.
- A single channel to the first convolutionary layer contains a time window for a single subband-electrode juxtaposition.
- It enables multi-class problem solving for pure EEG as opposed to image or other data types.

**Figure 2:**
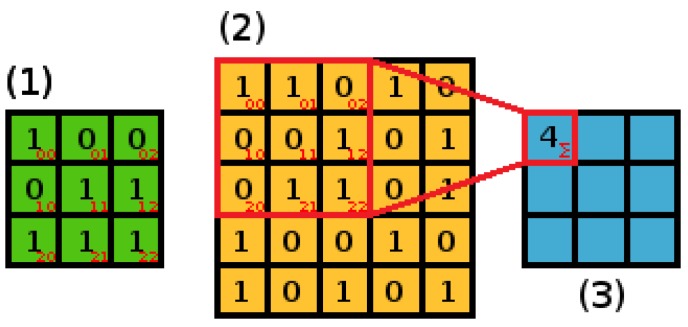
Calculation of the first component of a feature map (3), with a 3×3 filter (1) applied to 5×5 data (2). The red square within the input data (2) represents the actual region, where the filter (1) is applied to produce the first component of (3) according to Equation (1).

The characteristic element of a CNN is a convolution layer, which is similar to a perceptron layer but its task is to create a matrix of features. The convolution process involves calculating the dot product of a spatial region in the input data and an adaptable *filter* or *kernel* (Figure 2). The size of the filter should be adjusted to the structure of input data and the possible characteristics of considered input signal features, which is examined in the subsequent section of this paper.

(1) $$f\left(x,y\right)=\sum_{i,j=0}^{n}\left(a_{ij}\cdot b_{x+i,y+j}\right)$$

where f(x,y) is the output *feature map* element at position (x,y) of the data vector; $a_{i,j}$ is the element from the filter matrix; $b_{x+i,y+j}$ is the element from the spatial region of input data; *i*, *j* is the row and column index of current elements pair in the filter and *n* is the number of elements in the filter.

In the process of convolution, the filter (Figure 2, Matrix 1) is applied to different locations within the input data (Figure 2, matrix 2). The step size defining the relative position shifts of the filter is called a *stride*. The scalar produced for every coordinate pair (x,y) is stored in a *feature map* (Figure 2, Matrix 3) which represents the spatial distribution of input data features.

The calculation of the first element of a feature map for a data matrix size 5×5 and filter size 3×3 is presented in Figure 2. Turning now to Equation (1), Matrix 1 in Figure 1 refers to $a_{i,j}$ elements, the region of Matrix 2 marked red represents the $b_{i,j}$ elements, while the red numbers in right bottom corners describe indices *i* and *j*.

While analyzing the presented convolution process, a typical “single-channel” net may take into account information from many different spatial regions simultaneously. In image classification, this may include the information about the edge or color distribution, which is essential for effective classification of objects and scenes. In image processing tasks, splitting this information too finely may severely impair the obtained results. In the context of EEG recordings, we have to take into consideration that the signal is highly influenced not only by external sources of noise but also by the spatial characteristics of the data recording process. The electrodes positioned according to a standard 10-10 or 10-20 electrode placement system collect data which may be heavily distorted by the activity of the adjacent cortex areas—potentially irrelevant to the state of the user’s brain activity we want to classify.

Thus, for EEG data, our assumption is that the convolution of a single channel within a given time window can produce information that is more valuable and free of noise from other channels than if the signal is treated as a whole. With each of the multiple frequency channels analyzed independently, it is possible to better map a single electrode with respect to the potentially relevant frequency sub-band states.

**Figure 3:**
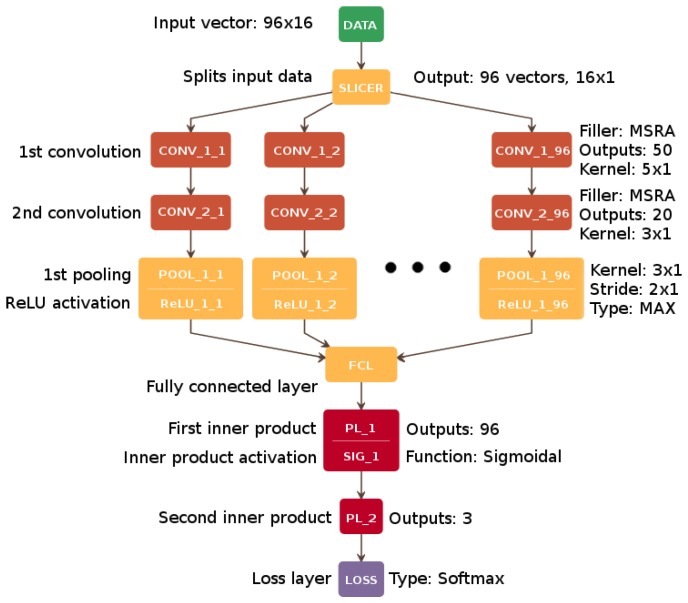
Multi-channel CNN architecture (CNN96). Architectural details are presented in Table 1.

The proposed architecture was initially developed as a single-channel solution (CNN1), which achieved a promising average classification rate of 64.50% on the BCI Competition dataset V. The final solution—CNN96—shown in Figure 3 and Table 1, with accuracy values presented in Section 6, is the result of a series of experiments and optimization of the learning process applied to the multi-channel implementation.

An enhanced multi-channel network architecture combined with using the whole vector of size 96×16 should prevent direct information mixing between individual channels, which is a drawback typical of single-channel networks. Thus, the most suitable overall number of convolutional channels was 96 (8 channels for each electrode multiplied by 12 frequency subbands: 8–10 Hz, 10–12 Hz, *…*, 28–30 Hz). The slicer input layer divides and provides the signal to convolutional layers. During this process, the signal is analyzed by every frequency band channel (96 channels—12 bands for each of 8 electrodes) represented by a single vector 16×1. It represents the time interval of 1 second by storing 16 consecutive points in time.

Competitive multi-channel solutions differ in such aspects as the role of convolutional layers as well as kernel size and features map generation. Cecotti dedicated the first hidden layer to time domain channel combination and the second hidden layer to subsampling and transforming the signal in the time domain [9]. Our network does not combine data from particular channels, processing them separately instead. In the first layer, it analyzes the frequency domain split into subbands, increasing the analyzed frequency resolution. In the second layer, it amplifies the frequency features maps with two consecutive convolutional layers. The difference lies also in the number of outputs. Cecotti’s architecture was designed for a binary output, whereas our solution is intended for a much more demanding multi-class problem.

In the architecture proposed here, two core stages of the signal analysis are specified. The first one involves noise reduction and amplification of neurological features stored in every channel which we assume to be correct in classification terms. The noise reduction is carried out by two consecutive convolution layers, to double the depth of feature filtering. Then, a pooling layer is applied using a max function to amplify the features previously filtered by convolution. We checked that only two of these layers presented good overall accuracy of classification and adding more of them did not improve the performance. The second stage involves composition and decoding. The former is performed with the fully connected layer, gathering and joining the channels, whereas the latter is carried out with just a single dense hidden layer with additional activation. The final number of hidden dense layers for the decoding phase was determined after a number of trials which proved that only one layer is sufficient to ensure good performance of the model.

Table 1 and Figure 3 contain the details of the CNN96 architecture. As mentioned previously, the convolution is carried out in every channel by two following CONV layers, with the proposed number of output connections equal to 50 and 20 in the first and the second layer, respectively. The initial weights of the filters were set with MSRA algorithm, which is more suitable for ReLU activation than sigmoid-like functions [61].

After these two convolutions, the MAX Pooling Layer (POOL_i) is added to reduce the dimensionality of the data by downsampling the data vector with a suitable kernel (filter) and stride. Next, the Fully Connected Layer (FCL_i) that connects the results from each previous convolutional layer is used. Two subsequent Perceptron Layers (PL_i), containing 96 outputs in the first layer and 3 outputs in the second one (one output per class), provide the input for accuracy evaluation within the Accuracy Layer (AL_i). AL calculates the correct classification of the current data vector and refers it to the accuracy threshold from the learning phase. Finally, the softmax loss (LOSS) function layer clamps and normalizes the output values.

In the present approach, the filters are initially set in one dimension (5×1 in the first phase and 3×1 in the second one) due to the slicing process where data provided to a single channel is a 16×1 vector. A single channel of data extracted in this way processes a single frequency band channel retrieved from one electrode [14].

The initial parameters of the CNN96 architecture were further optimized, which additionally boosted the effectiveness of the network. More details on this are given in Section 5.

To compare CNN96 with other well-known architectures, we selected two of the most successful single-channel architectures (AlexNet and VGG-16) [12,16] for image classification tasks and prepared the multi-channel variations of their first two convolution stages (for variation preparation, each consecutive convolution layer was treated as a single stage). The main reason for this was to check if the competitive architectural solutions implemented in these networks could contribute to improving the accuracy of mental tasks classification.

The development process of these variations was restricted to the initial convolutional layers because of the relatively small size of the input data vector. If the whole complexity of AlexNet and VGG-16 architectures were used, the further stages could result in poor filter learning performance, which might lead to a generalization error.

**Figure 4:**
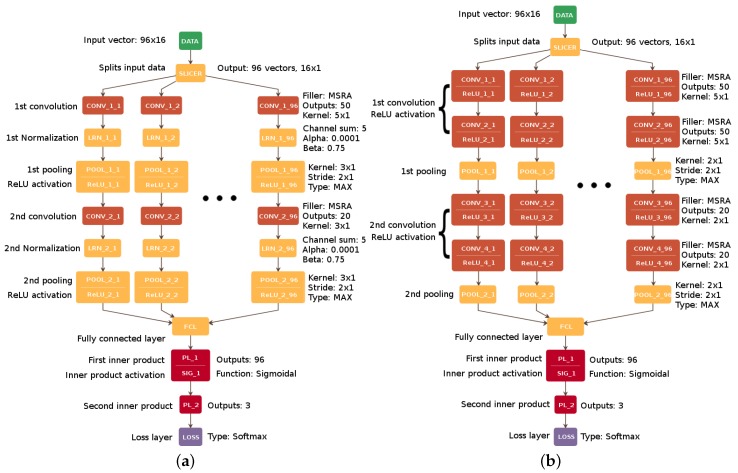
The AlexNet multichannel variation developed for the architecture comparison and performance analysis (**a**); and the VGG-16 multichannel variation developed for the architecture comparison and performance analysis (**b**).

While the AlexNet variation presented in Figure 4 uses a normalization layer based on the Local Response Normalization operation ($LRN_{i}$ layer) to improve network generalization [16], VGG-16 makes use of several consecutive convolution layers stacks with small kernel sizes to avoid overfitting [12] and to focus on the recognition of small patterns rather than more complex ones.

**Figure 5:**
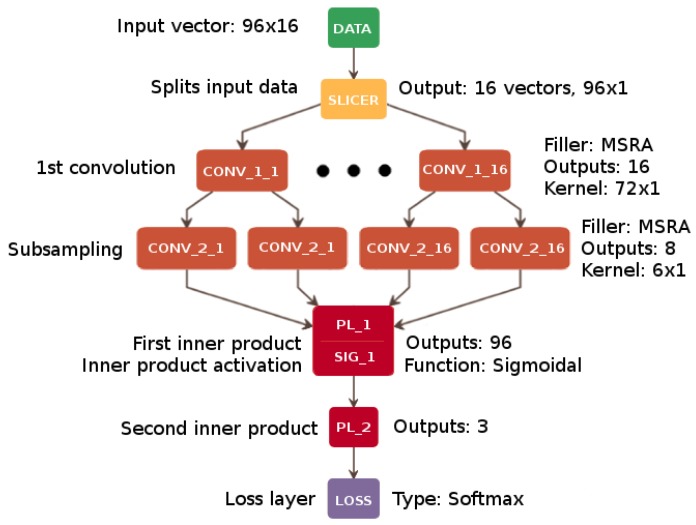
The adaptation of Cecotti’s multi-channel network architecture to our problem.

For the purpose of the present study, we also reconstructed the topology of the reference solution [9], adapting it to the applied dataset (Figure 5). For network parameters such as the fill function for weights initialization and any other parameters with values unspecified by Cecotti, we assumed the most promising values found during the optimization of the proposed method.

## 5. Optimization of the Learning Process

To further improve the performance of the CNN96 network, we conducted a series of optimization tests for both effectiveness/accuracy improvement (*ACCOP* phase) and learning rate optimization (*LFMOD* phase). For the *ACCOP* phase, to enhance the learning process and the resulting final accuracy, we assumed two separate areas of improvement:

- manipulation of layers base functions parameters, such as the number of outputs and kernel size (*PARMOD* phase); and
- testing selected learning rate modification functions for more training flexibility (*LEARNOP* phase).

During the optimization of the learning rate, the following parameters were applied to the learning functions (*lr*): initial learning rate (*baseLr*) = 0.01, *gamma* = 0.75, *stepsize* = 500, iteration number (*maxIter*) = 15,000 and *power* = 2. The functions are given as follows:

(2)lrFIXED(i)=baseLr(3)lrSTEP(i)=baseLr·gamma⌊istepsize⌋(4)lrEXP(i)=baseLr·gammai(5)lrPOLY(i)=baseLr·(1−imaxIter)power(6)lrSIGMOID(i)=baseLr·11+e−gamma·(i−stepsize).

where ⌊·⌋ is the floor function.

The final values of the variables selected for optimization were determined based on the highest accuracy results obtained from tests, as presented in the following sections.

### 5.1. PARMOD Phase

The main goal of the PARMOD phase was to find an appropriate parameter setting for the network core layers to further improve the learning progress. We selected the most significant parameters for learning—the base size of the first convolution filter: 5×1 (CONV1); the base size of the second convolution filter: 3×1 (CONV2); and the number of outputs of the first perceptron layer: 200 (PL1)—to test if the modifications of these values would increase the classification accuracy within a fixed number of iterations. All of the base values of these parameters were initially set by empiric choice and then carefully modified to suit the purpose of our tests. For each of the parameters, four test values were calculated with respect to its base (initial) value, with a 25% and 50% increase and decrease (rounding up to the nearest higher integer):

(7) $$p_{\Delta }\left(x\right)=\left\lceil p\left(x+x\cdot \Delta \right)\right\rceil$$

where $p_{\Delta }$ is the modified parameter calculated from *x*; *x* is the value of the parameter to be modified; Δ is the modification factor where Δ∈{−0.50,−0.25,0.25,0.50} and ⌈·⌉ is the ceiling function.

### 5.2. LFMOD Phase

In the search for the best strategy for the learning rate change during the training process, we performed several tests using selected available options. The learning rate is an important factor that controls the rate of changes of the neural weights’ values in the course of training. If set too small, it will result in the learning process being slow and ineffective. With the learning rate set too high, the optimization goal may not be met with sufficient precision and the training process may become unstable. Therefore, it is typically set high at the beginning and then gradually reduced during training, according to a monotonically decreasing function. In the LFMOD phase, we tested five functions to find the one for which the net would demonstrate the greatest learning progress: fixed (constant), step (linear), exponential, sigmoid and polynomial functions.

### 5.3. LEARNOP Phase

Besides the new architecture of the CNN96 network, an additional contribution of the present study lies in the adaptive elaboration of the applied filter characteristics. The filters are used to process the input data vector with a discrete stride value. The results of filter calculations for a given layer act as a matrix of features which correspond to the output weights of the neurons subjected to the activation function. Due to the complex characteristics of EEG signal and its still poorly known correlations of features, the weight adaptation algorithms that depend on gradient error history rather than on momentum were applied to the final accuracy and training progress results.

By choosing a proper solver type, we address the general optimization problem during network update from learning accuracy by minimizing the loss factor to memory complexity. The Adaptive Gradient [55], similar to SGD [56], is a gradient-based optimization method for computing the necessary parameter updates, but in fact it is not based on momentum. This method, similar to other algorithms based on parameter history, does not rely on the momentum since it does not involve speeding up the training per-dimension, computing the norm of the previous gradients instead. These types of methods use the history of previous gradient updates to predict the most valuable update of actual weights matrix state. The weights update method proposed by J. Duchi is as follows:

(8) $${\left(W_{t+1}\right)}_{i}={\left(W_{t}\right)}_{i}-\alpha \frac{{\left(\nabla L\left(W_{t}\right)\right)}_{i}}{\sqrt[{}]{\sum_{t^{\prime }=1}^{t}{\left(\nabla L\left(W_{t^{\prime }}\right)\right)}_{i}^{2}}}$$

where *t* is the current iteration number; *i* is the actual component of weights *W*; *W* is the state of previous weights; ${\left(W_{t+1}\right)}_{i}$ is the updated weights state in the following (*t* + 1) iteration of *i* component; α is the global learning rate shared by all dimensions; ${\left(\nabla L\left(W_{t^{\prime }}\right)\right)}_{i}$ is the norm of previous gradients on a per-dimension basis; and $t^{\prime }$ is the previous iteration number ($t^{\prime }\in \left\{1,2,\ldots t\right\}$) [55].

## 6. Results

### 6.1. PARMOD Phase

Table 2 contains the average change of the achieved accuracy level (rounded to integer values) over the training session, as compared to the session with base parameters from the CNN1 architecture. Value 0% means that no difference in the learning progress was noticed. The decrease of the CONV2 filter size by 25% and 50% is not included because it resulted in an inability to train the network.

### 6.2. LFMOD Phase

**Figure 6:**
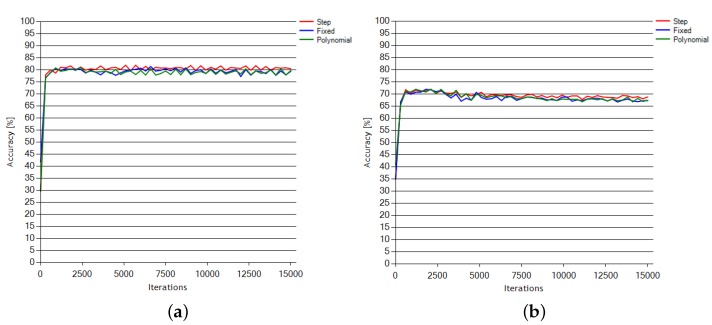

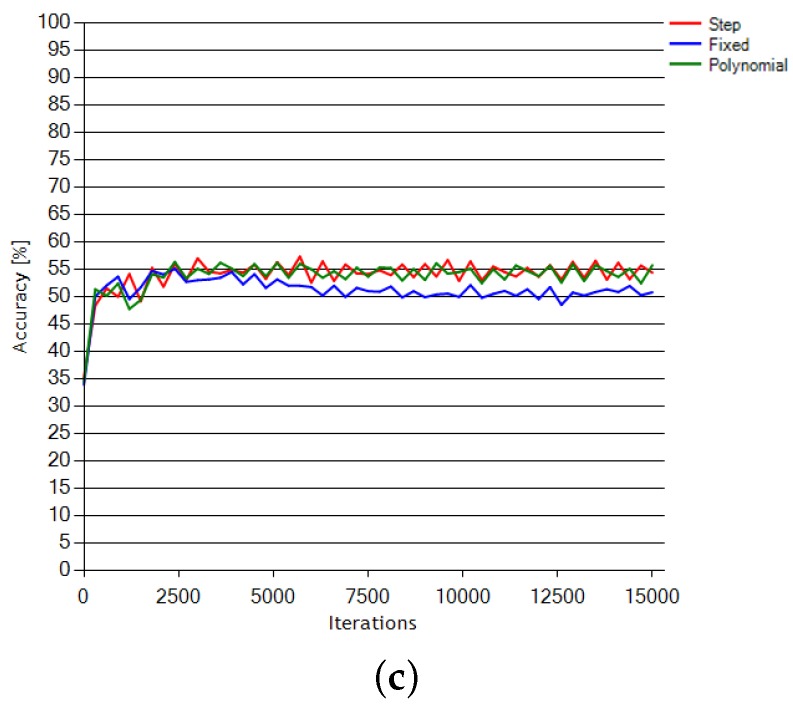
(**Top**) Accuracy change over iterations for Subjects 1 and 2 dataset training; and (**Bottom**) accuracy change over iterations for Subject 3 dataset training.

**Figure 7:**
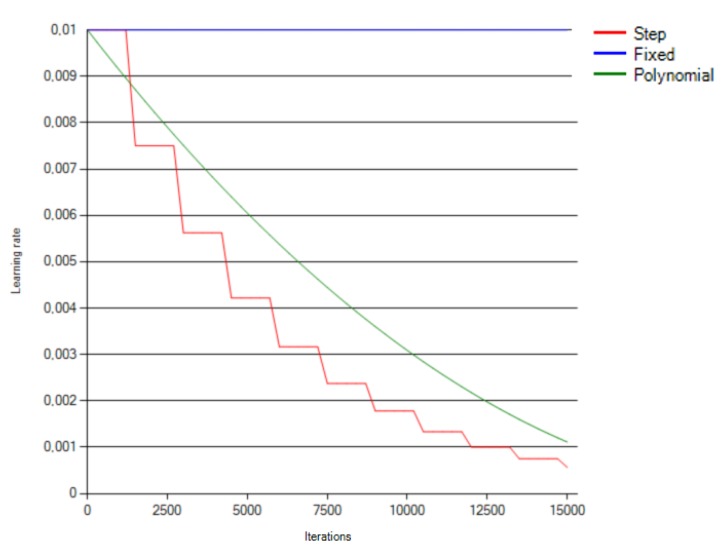
Learning rate over iterations based on the same learning rate functions as those in Figure 6.

The tests performed during the LFMOD phase, including the results for step, exponential and fixed learning rate functions, are presented in Figure 6 and Figure 7. Figure 6 presents the accuracy progress over all three subjects learning sessions, corresponding to the learning rate change depicted in Figure 7.

The exponential and sigmoid functions demonstrated a negligible change in the learning progress and, therefore, have been excluded from the above presentation.

### 6.3. LEARNOP Phase

**Figure 8:**
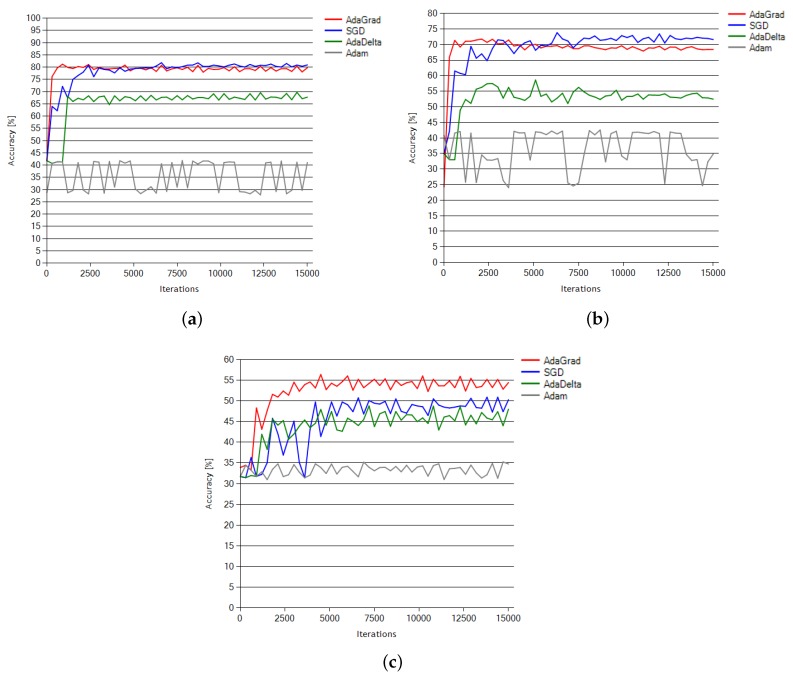
(**Top**) Learning curves for Subject 1 and 2 dataset training; and (**Bottom**) learning curves for Subject 3 dataset training.

The calibration process of hyperparameters learning was verified against modern stochastic optimization solvers: AdaGrad [55], SGD [56], AdaDelta [57] and Adam [58]. AdaGrad proved most effective in terms of the learning speed and accuracy with the net tested against three separate subjects from the dataset presented in [14]. The calibration covered both time and accuracy level over certain amounts of learning time (5000, 10,000 and 15,000 iterations). Using the AdaGrad algorithm, the CNN96 architecture (Figure 3) achieved the best average results as compared to other NN architectures tested on the same dataset. The learning curves based on the training sessions of all four algorithms to the maximum of 15,000 iterations are presented in Figure 8.

The SGD algorithm was included in the comparison as a typical example of momentum-based methods and a representative of non-adaptive methods. During the optimization tests, this method reached a 1–2% higher accuracy level than AdaGrad with Subject 1 dataset (Figure 8a) and a 4% higher accuracy level than AdaGrad with Subject 2 dataset (Figure 8b).

### 6.4. Effectiveness and Generalization Errors

The effectiveness and generalization errors generated by our method are presented in Table 3 and Table 4, respectively. The results are given for 8-sample (0.5 s) and 16-sample (1 s) variants. The tables include separate data for the subjects and calculated average values.

### 6.5. ROC Analysis

Table 5 shows the confusion matrices for the multi-class problem according to Labatut and Cherifi [62], where the effectiveness, or *overall success rate* (**OSR**), is defined as the trace of the confusion matrix, divided by the total number of classified instances. It is multi-class, symmetrical, and ranges from 0 to 1. For example, for Subject 1, it is equal to $\frac{905+725+1222}{3504}=0.8140$, where 905 is the number of instances belonging to Class 2 classified correctly as Class 2, 725 is the number of instances belonging to Class 3 classified correctly to this class, and 1222 concerns Class 3 in the same way. These numbers are presented in Table 5 under the title “Subject 1”. The total number of classifications is 3504. The example of OSR for Subject 1 is shown in Table 3 as the effectiveness of CNN96-16sam for Subject 1.

To calculate common ROC parameters, the multi-class problem should be presented as binary class–non-class one. Table 6 presents an example of binary approach to Class 2 and Subject 1.

Table 7 shows the parameters calculated for all three subjects and all three classes for each of them.

## 7. Discussion

### 7.1. Optimization Phases

The PARMOD phase was concerned with testing the accuracy progress. As can be seen in Table 2, the manipulation of the first convolution filter size resulted in the decreased accuracy in all cases. The only relevant case of the second convolution filter size manipulation did not change the learning, so it could only potentially decrease the time complexity of the learning process. From the results illustrated in Table 2, we can also see that the reduction of the number of the PL layer outputs did not affect the accuracy of the net, even when the values were doubled down. On the other hand, the increased values resulted in worse accuracy.

The LFMOD tests proved that the step (linear) learning function was an optimal selection (Figure 6) in terms of the learning progress. The sigmoid function resulted in the net starting to learn with a noticeable delay, depending on the session. Fixed, exponential and polynomial functions resulted in a minimal progress change or a noticeable decrease of the accuracy progress.

The LEARNOP phase aimed to examine the calibration process of hyperparameters learning. Although the training sessions of all four algorithms provided 15,000 iterations, the best results were achieved within 5000 iterations (Figure 8). Therefore, under the presented experimental conditions, the 5000-iteration interval may be considered as the optimal choice to avoid overtraining. Additionally, the SGD algorithm achieved slightly better results here. However, it should be mentioned that these results emerge from training progress over about 6000 iterations, which is still above the optimal results for AdaGrad. The momentum-based method used by the SGD algorithm [56] relies directly on the last update of the weights (“short history” of updates). In this situation, the actual gradient update converges following the summed up vector of the previous updates.

### 7.2. Comparative Analysis of CNN Architectures

For performance comparison, all architectures were tested on dataset V from BCI Competition III [14]. It contains data acquired from three individuals (Subjects 1, 2 and 3). For every subject, the data consisted of three training files and one testing file. All of the results presented were calculated as an average of five repeats with the same NN configuration to minimize the randomization error. The effectiveness value was calculated as a percentage of correct pattern classification against the labels provided as the 97th component in the testing file. As compared with the single-channel solution, the multi-channel approach resulted in an improvement of 2–8%. The classification results differed quite significantly depending on the subject (Table 8). The CNN96-16sam and CNN96-8sam represent our solution using 16 and 8 consecutive samples in time, respectively. The shorter time window was tested following the specification of BCI Competition III, which imposes a 0.5-s interval to guarantee a fast response time. The longer (1-s) time window was used under the assumption that it was more suitable for mental state analysis than 0.5-s blocks. Wider time windows were not tested because of too long a response time for real-time calculation systems that the method is intended for. Another assumption was that time windows longer than 1 s would be likely to affect the analysis results due to the presence of too much noised data. We observe in Table 8 that the most comparable multi-channel solution using two layers of convolution—Cecotti—achieved much worse results due to the maladjustment of architecture to the problem under examination. The reason for the poor performance of Cecotti’s method is threefold. First, the problem requires a lot of flexibility when it comes to the number of EEG electrodes used. Secondly, initial processing involving the exchange of input from the time domain to the frequency domain is required to provide better results. Thirdly, our method is intended to deal with a multi-class problem, whereas Cecotti’s solution is designed for single-class P300 detection.

The solutions were also examined for generalization errors, as many authors reported considerable differences in classification accuracy during the training process and dataset testing. The generalization error reflects the adaptability of the learned classifier to a new, unknown dataset. Table 4 presents generalization errors measured for the proposed solution considering 8 or 16 samples.

Table 9 presents the results of the two variations of AlexNet and VGG-16 architectures compared to those obtained by the CNN96 method proposed in this paper. As we can see from the results, the accuracy achieved by our method is slightly but significantly higher than the values reached by the other architecture variations. The most noticeable difference in accuracy values, which is observed for Subject 3 dataset, suggests that noise reduction provided by single consecutive convolution layers is more efficient in handling highly noised signal recordings than the solutions proposed in the variations of multiple activations (VGG-16) or multiple normalization (AlexNet). The similarity of results obtained for Subjects 1 and 2 proves the assumption that convolution layers can be applied as a good noise reduction mechanism, even for complex models. On the other hand, higher values of CNN96 can suggest that, in the case of EEG signal, neither multiple response normalization nor stacked activation convolutions are as good as proper feature amplification by a single max pool layer presented in our approach. However, the results are not sufficient to definitely conclude whether the specific features of the variations, such as local response normalization or multiple consecutive stacked convolutions with additional ReLU activation, would be valuable for our method.

## 8. Conclusions

To evaluate the contribution of this work in a broader context, we compared our results with three other methods: Cecotti’s multi-channel architecture and multi-channel variations of AlexNet and VGG-16. Under the presented experimental conditions, the results obtained by our method compare favourably to those achieved by the other solutions. During the learning optimization stages, we managed to noticeably increase the classification accuracy within a reasonable iteration count needed for learning. Demonstrating a remarkable generalization rate of about 5%, our multi-channel approach, supported with signal frequency band splitting analysis, is currently the most competitive solution for mental tasks classification.

The comparison of the CNN1 and CNN96 architectures is presented along with other NN methods tested against the same dataset. Although SGD optimization solver performed well with Subjects 1 and 2, it was still below the comparison expectations for AdaGrad in the case of Subject 3, which appears to be the most problematic for all the classification methods. The proposed approach based on the presented CNN architecture enhanced with adaptive gradient (AdaGrad) optimization outperforms other NN-based methods used for mental tasks classification.

Current network solutions demonstrate effectiveness ranging from about 58% to about 68%. This is a small scatter and the final result depends on minor changes in architecture and learning. With effectiveness results of up to 70%, our solution outperforms the other methods.

ROC analysis was applied to evaluate the classifiers and confirm the reliability of our results.

To conclude, the results of this study are very encouraging. However, in the field of mental task classification and overall EEG analysis for BCI usage, there is still abundant room for further progress. Therefore, further research is recommended to investigate the full potential of multi-channel, selective network approaches and evaluate their usefulness for practical applications. 

## Figures and Tables

**Table 1:** CNN96 architecture description.

Layer	Layer Type	# Filters	Size	# Params	Output Dimension	Activation	Mode
1	Input	-	-	-	(96, 16)	-	-
	Slice	-	-	-	(1, 96, 16)	-	-
	Conv1D	50	(1, 5)	96 × 50	(50, 96, 16)	Linear	valid
2	Conv1D	20	(1, 3)	96 × 20	(20, 96, 16)	Linear	valid
	Pool	-	(2, 1)	1 × 20	(10, 96, 16)	-	-
	Activation	-	-	-	(10, 96, 16)	ReLU	-
3	FullyConnected	-	-	-	96	-	-
	InnerProduct	96	-	96	96	Linear	-
	Activation	-	-	-	96	Sigmoid	-
4	InnerProduct	96	-	96	3	Linear	-
	Loss	-	-	-	-	-	-

**Table 2:** Net parameterization results obtained from PARMOD tests—the average training change for all the three subjects from the examined dataset.

Δ Values and Corresponding Network Parameters	Average (Rounded) Accuracy Progress Change Over Training (%)
−50%—CONV1 filter size (3×1)	−20%
−25%—CONV1 filter size (4×1)	−2%
+25%—CONV1 filter size (7×1)	−2%
+50%—CONV1 filter size (8×1)	−2%
+25%—CONV2 filter size (4×1)	0%
+50%—CONV2 filter size (5×1)	0%
−50%—PL1 outputs (100)	0%
−25%—PL1 outputs (150)	0%
+25%—PL1 outputs (250)	0%
+50%—PL1 outputs (300)	−2%

**Table 3:** Effectiveness of the method (in %).

Effectiveness	Subject 1	Subject 2	Subject 3	Average
CNN96-16sam	81.40	72.10	55.70	69.73
CNN96-8sam	79.90	68.53	53.05	67.16

**Table 4:** Generalization errors for the examined subjects (in %).

Generalization Error	Subject 1	Subject 2	Subject 3	Average
**CNN96-8sam**	5.04	4.54	11.59	**7.05**
**CNN96-16sam**	2.79	2.16	10.73	**5.22**

**Table 5:** Confusion matrices for the three subjects.

	Subject 1				Subject 2				Subject 3			
		2	3	7		2	3	7		2	3	7
predicted class	2	905	213	61	2	532	112	223	2	263	114	104
	3	64	725	157	3	183	917	178	3	592	875	211
	7	71	86	1222	7	149	123	1055	7	345	149	805

**Table 6:** Confusion matrix for Subject 1 and Class 2.

		Actual Class		
	**Total = 3504**	**2**	**Non-2**	
predicted	2	905	274	PPV = 0.7676
class	Non-2	135	2190	NPV = 0.9419
		TPR = 0.8702	TNR = 0.8888	ACC = 0.8833

**Table 7:** ROC parameters for all three subjects and all three classes for each of them.

Subject	OSR	Class	ACC	TPR	TNR	PPV	NPV
1	0.8140	2	0.8833	0.8702	0.8888	0.7676	0.9419
		3	0.8516	0.7080	0.9109	0.7664	0.8831
		7	0.8930	0.8486	0.9239	0.8861	0.8974
2	0.7216	2	0.8084	0.6157	0.8722	0.6150	0.8726
		3	0.8288	0.7974	0.8444	0.7175	0.8937
		7	0.8061	0.7246	0.8649	0.7950	0.8129
3	0.5571	2	0.6603	0.2192	0.8916	0.5147	0.6853
		3	0.6858	0.7491	0.6539	0.5215	0.8381
		7	0.7681	0.7188	0.7914	0.6197	0.8561
mean	0.6975		0.7984	0.6946	0.8491	0.6893	0.8535
standard deviation	0.1301		0.0812	0.1943	0.0830	0.1284	0.0731

**Table 8:** Effectiveness comparison of the ANN-based methods (in %). The Cecotti * row presents the results obtained using Cecotti’s architecture and feeding. The Galan ** row presents the results obtained by the statistical discrimination-based method that won the international BCI Competition III.

Method	Subject 1	Subject 2	Subject 3	Average
Cecotti *	44.90	39.02	32.11	38.68
GANN	69.32	66.32	44.40	58.01
BPNN	76.02	65.89	51.14	64.34
CNN1	78.22	62.80	52.49	64.50
PSONN	75.98	69.78	53.83	66.33
2DCNN-big	80.74	67.89	52.98	67.20
IPSONN	78.31	70.27	56.46	68.35
**CNN96-8sam**	**79.90**	**68.53**	**53.05**	**67.16**
BSANN	80.32	66.03	59.34	68.56
Galan **	79.60	70.31	56.02	68.64
**CNN96-16sam**	**81.40**	**72.10**	**55.70**	**69.73**

**Table 9:** Results of AROP phase tests using AlexNet variation (Figure 4a) and VGG-16 variation (Figure 4b).

Architecture Variation	Subject 1	Subject 2	Subject 3	Average
AlexNet	79.48	68.48	53.84	67.26
VGG-16	79.45	71.77	53.50	68.24
**CNN96-16sam**	**81.40**	**72.10**	**55.70**	**69.73**
